# Ovarian Angiosarcoma With Intractable Intraperitoneal Hemorrhage: A Case Report and Review of the Literature

**DOI:** 10.7759/cureus.76849

**Published:** 2025-01-03

**Authors:** Naoki Tsuchiya, Yuichi Imai, Tamaki Cho, Yuki Ogawara, Taichi Mizushima, Hiroki Takase, Satoshi Fujii, Etsuko Miyagi

**Affiliations:** 1 Obstetrics and Gynecology, Yokohama City University Graduate School of Medicine, Yokohama, JPN; 2 Molecular Pathology, Yokohama City University Graduate School of Medicine, Yokohama, JPN

**Keywords:** intraperitoneal hemorrhage, literature review of disease, next generation sequencing (ngs), primary ovarian angiosarcoma, therapy

## Abstract

Primary ovarian angiosarcoma is an extremely rare malignant soft tissue tumor that arises from the vascular endothelium, with a limited number of reports. It has no specific symptoms, and it is rarely diagnosed in the early stages because of rapid progression. Furthermore, no standard drug therapy is available, and the prognosis is poor.

The patient was a 68-year-old woman suspected of having a ruptured ovarian tumor on plain abdominal computed tomography and anemia was observed. An emergency laparotomy was performed to stop the bleeding. Thereafter, one transcatheter arterial embolization, three laparotomies, and local radiotherapy were performed in an attempt to achieve hemostasis. However, the intraperitoneal bleeding remained intractable, and the transfusion was terminated after thorough consultation with the patient. The patient died 44 days after admission. Autopsy findings confirmed the diagnosis of primary ovarian angiosarcoma and pulmonary metastasis.

Intractable intraperitoneal hemorrhage similar to that observed in this case has been reported, and because the prognosis is extremely poor, establishing an appropriate treatment regimen is desirable. Previously, the most commonly used drug regimens included anthracyclines and ifosfamide. However, recently, the possibility of treatment with molecular targeted agents and immune checkpoint inhibitors has been reported. In the future, case accumulation and genetic analyses will be required to establish appropriate treatment methods.

## Introduction

Angiosarcoma (AS) is a malignant soft tissue tumor that arises from the vascular endothelium. The incidence of malignant soft tissue tumors is approximately 1.5% of all malignant tumors [1]. AS is extremely rare, accounting for less than 2% of malignant soft tissue tumors, and has high local invasion and metastasis potential, with a poor prognosis [1]. It can develop in any organ, including the skin, soft tissue, breast, liver, and bone [2]. Primary ovarian AS (OAS) is extremely rare, with only 47 cases reported in the English literature to date. Although a theory that AS is caused by DNA damage in the cells of blood or lymphatic vessels due to radiation or trauma exists, the exact cause of AS remains unknown. Primary OAS is usually unilateral, premenopausal, and sometimes associated with teratomas; more than half of the cases are diagnosed in stage III or IV [3,4]. As with other types of AS, the disease progresses rapidly, and because it is rare, no treatment has been established to ensure long-term survival; thus, the prognosis remains very poor.

We encountered a case of primary OAS with intractable hemoperitoneum, resulting in the death of the patient after approximately one and a half months of treatment. We discuss the case in relation to the treatment, prognosis, and nature of the tumor and a review of the existing literature.

## Case presentation

**Figure 1:**
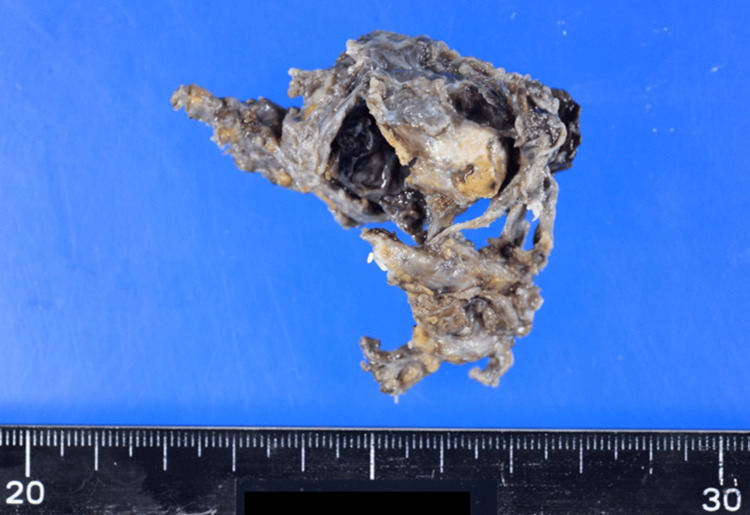
Removed right ovarian tumor The tumor was located anterior to the right ureter, medial to the right internal iliac artery, in continuity with the pelvic infundibular ligament, and was judged to be a right ovary. The tumor was found to be partially ruptured and hemorrhaging.

The patient was a 68-year-old woman (gravida 0) who had a history of undergoing total abdominal hysterectomy for uterine myoma (24 years prior) and laparoscopic appendicectomy for appendicitis (10 years prior). There was no noteworthy family history or comorbidities. The patient was diagnosed with anemia during blood donation and had mild lower abdominal pain at the time of presentation. Investigations performed per the advice of the local doctor revealed a serum hemoglobin level of 7.8 g/dL, a right ovarian tumor measuring approximately 4 cm, and severe ascites on plain abdominal computed tomography (CT), suggesting rupture of the tumor. Subsequently, the patient was referred to our hospital. Since no increase in the volume of ascites was observed after admission and the lower abdominal pain was relieved by symptomatic treatment, we decided to conduct a close examination on a wait-and-see basis. On the fourth day of hospitalization, owing to a decrease in serum hemoglobin level and an increase in the volume of ascites that were not commensurate with the amount of concentrated red blood cells transfused, we performed an emergency laparotomy to achieve hemostasis. The pelvic cavity showed severe adhesions due to previous surgeries. Although the left adnexa was not visible, the right ovary was found to be partially ruptured and hemorrhaging (Figure 1).

**Figure 2:**
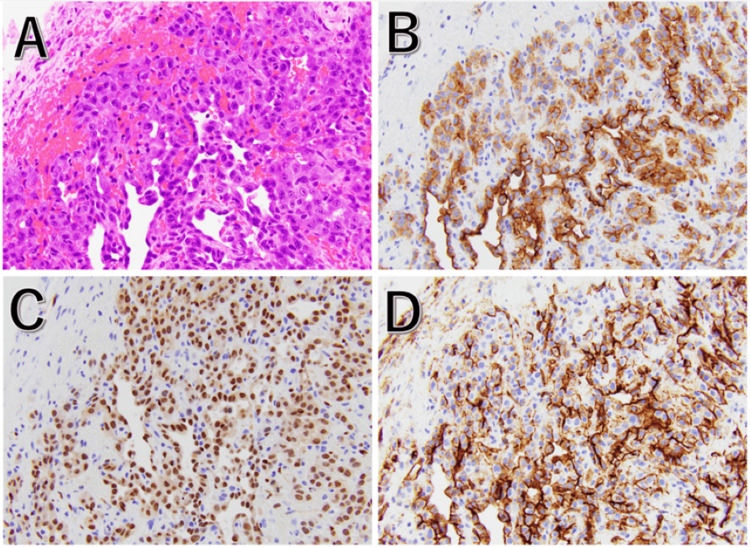
Pathological findings The tumor is observed to have developed contiguously from the ovarian parenchyma. The vascular endothelium is displaced and occupied by the tumor cells. Each tumor cell includes well-defined, round nucleoli, an eosinophilic endoplasmic reticulum, and a highly atypical nucleus. Angiosarcoma was diagnosed based on the presence of vascular endothelial markers CD31, ERG, and CD34. (A) Hematoxylin-eosin stain, (B) CD31, (C) ERG, and (D) CD34. This pathology image is at 200x magnification.

No disseminated nodules were observed in the abdominal cavity. The patient was diagnosed with hemoperitoneum due to the rupture of the right ovarian tumor, and a right salpingo-oophorectomy was performed. The tumor was histopathologically diagnosed as primary OAS. The basis for the diagnosis is “multilayering of endothelial cells, nuclear atypia, increased mitoses, necrosis” and “positive for vascular markers, such as CD31, CD34, ERG, VEGF and factor VIII, by immunohistochemistry” (Figures 2A-2D).

**Figure 3:**
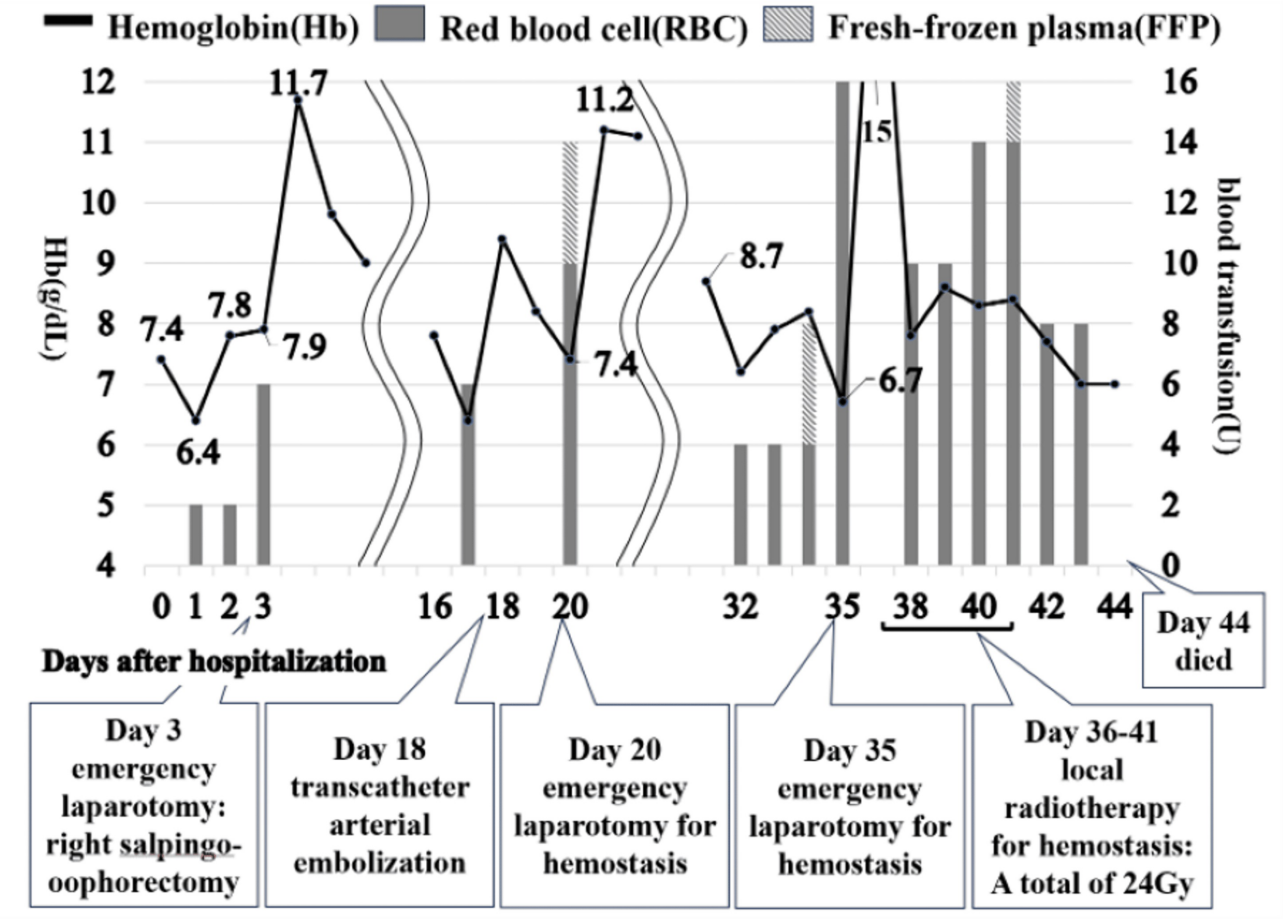
Clinical progress This figure shows trends in serum hemoglobin levels in relation to the blood transfusions received.

Postoperatively, hemostasis was temporarily achieved, but the serum hemoglobin level gradually decreased from 15 days after hospitalization and reached 6.4 g/dL after 17 days of hospitalization. Hemoperitoneum reappeared on abdominal contrast-enhanced CT. Extravasation was observed from a branch of the right internal iliac artery, and transcatheter arterial embolization was performed. Serum hemoglobin level continued to decline; a second laparotomy was performed 20 days after hospitalization, and a third laparotomy was performed 35 days after hospitalization. Because there was a possibility of hemostatic radiation to the site of the right ovarian tumor removal, a clip was placed at the same site at the third laparotomy to mark the site for radiation. Intraoperatively, dark brown peritoneal seeding nodules were observed in the abdominal cavity, which rapidly increased and showed a tendency to increase. Since the patient wanted to be treated to the greatest extent possible, we initially considered chemotherapy for AS but made the decision to control bleeding first and, therefore, administered local radiotherapy (total dose, 24 Gy) to the right adnexal region, which was the source of bleeding. However, the intraperitoneal bleeding remained intractable, with a daily bleeding rate of 1,200 to 1,500 mL, and after thorough consultation with the patient, the transfusion was terminated. The patient died 44 days after admission without achieving hemostasis (Figure 3).

**Figure 4:**
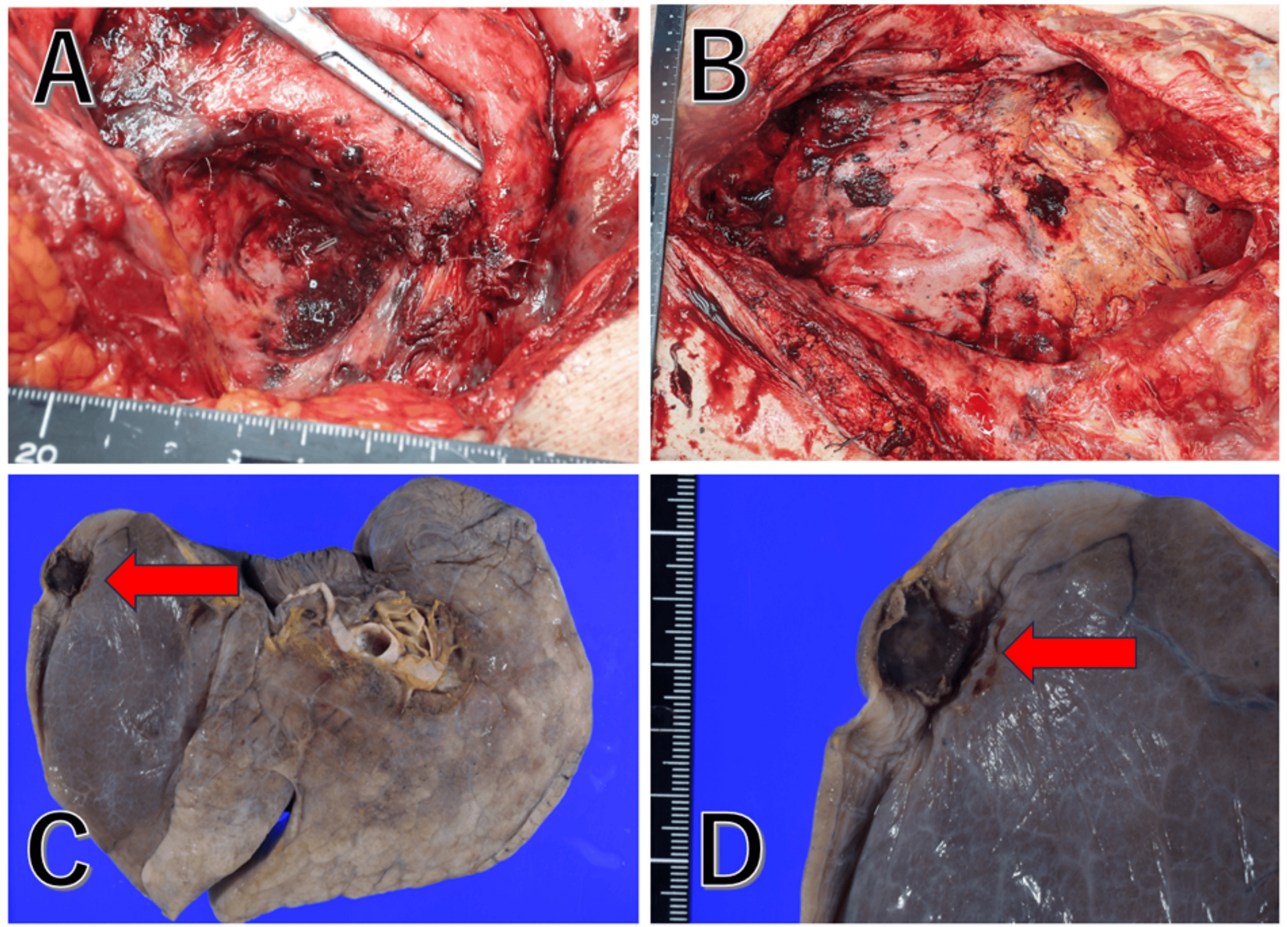
Autopsy findings (A) Right salpingo-oophorectomy site. (B) Entire intraperitoneal region. The peritoneal seeding nodules are centered on the right salpingo-oophorectomy site and extended into the intraperitoneal region. (C, D) Left lung metastasis A dark brown nodule, 2 cm in diameter, is seen (red arrows). No evidence of mediastinal metastases is seen, indicating hematogenous metastasis.

Pathological autopsy revealed that the abdominal cavity was filled with a large amount of hemorrhage (approximately 5,200 mL), and the peritoneal cavity was widely scattered with dark brown disseminated nodules. In addition, nodules similar to those in the peritoneal cavity were present on the pleural surface of the lungs. Since there were no disseminated nodules in the thoracic cavity or on the thoracic surface of the diaphragm and some pathological findings for vascular invasion, the diagnosis of hematogenous metastasis was made (Figures 4A-4D). Pre-clipping and irradiation of the right ovarian tumor excision were performed, but there were no histopathological changes, and the effect of treatment was not evident.

## Discussion

This paper is a literature review of a total of 48 cases, adding this case to the past primary OAS, and discusses the largest number of cases to date in terms of their number and characteristics in terms of treatment (Table 1) [5-38].

**Table 1:** Previously reported cases of ovarian angiosarcoma AI: doxorubicin, ifosfamide, MAID: Mesna, doxorubicin, ifosfamide, dacarbazine, DG: docetaxel gemcitabine, DOD: dead of disease, AWD: alive with disease, NED: no evidence of disease Details on olaparib, apatinib, and nivorumab are provided in Table 2.

Year	Author	Reference no.	Age	Symptoms	Staging	Primary treatment	Adjuvant treatment	Follow-up (months)	Status	Special notes
1982	Ongkasuwan C	5	77	Abdominal distension	III	USO	None	2	DOD	With mucinous cystadenoma post menopause
1991	Patel T	6	42	Abdominal pain	IV	USO	None	<1	DOD	
1994	Cunningham MJ	7	19	Abdominal discomfort	IV	USO+OMT+LND	AI	7	DOD	
1996	Nara M	8	33	Hemoptysis	IV	Lung biopsies	None	2	DOD	
1997	Nielsen GP	9	20-32 Average: 26	Abdominal pain	I	NR	NR	66	NED	
				Abdominal pain	I			108	NED	
				Abdominal pain	I			NR	NR	
				Abdominal pain	I			NR	NR	With dermoid
				Abdominal pain	III			2	DOD	With dermoid
				Abdominal pain	III			15	DOD	
				Abdominal pain	III			30	DOD	
1998	Furihata M	10	46	Abdominal mass, abdominal discomfort	Estimated I	TAH+BSO+LND	Cysplatine＋palaaorta radiationation	9	DOD	
1998	Lifschitz-Merce Br	11	25	Abdominal pain	III	USO+OMT+LND	AI	18	DOD	
1998	Nucci MR	12	35	Palpable ovarian mass	IV	USO	None	1	DOD	
			42	Hemoperitoneum	I	USO	NR	24	DOD	
			27	Abdominal pain	I	USO	None	14	NED	
			25	Abdominal pain	III	USO	None	3	NED	
1999	Jylling AM	13	37	Cyst	I	USO	NR	NR	NR	With mucinous cystadenocarcinoma
1999	Platt JS	14	40	Abdominal pain	IV	Suboptimal debulking	MAID	NR	NR	
1999	Twu NF	15	38	Hemoptysis	IV	Full staging debulking	AI	7	DOD	Bilateral
2001	Pillay K	16	45	Abdominal distension	IV	Suboptimal debulking	None	3	DOD	With borderline serous cystadenocarcinoma
2005	Davidson B	17	19	Abdominal pain	III	USO	AI+radiation	12	DOD	
2005	Jha S	18	28	Abdominal pain	I	USO	Secondary debulking surgery AI	72	NED	
2005	Quesenberry CD	19	31	Abdominal distension	IC	Full staging debulking	MAID	10	NED	
2006	den Bakker MA	20	30	Abdominal pain	IIIC	Debulking	Bleomycin etoposide cisplatin	9	DOD	With dermoid
2009	Contreras AL	21	32	Bloating, abdominal pain	IV	TAH+BSO+OMT+apendectomy	AI	29	DOD	With dermoid
2010	Bradford L	22	67	Bloating fatigue, abdominal pain	IIIC	Full staging debulking	None	1	DOD	Postmenopause
2010	Cambruzzi E	23	65	Sensation of heaviness in the hypogastrium	IIIC	TLH	Chemotherapy (not otherwise specified)	2	NED	With fibroma postmenopause
2010	Serrano C	24	23	Abdominal pain	IV	Suboptimal debulking	Epirubisin ifosfamide	12	NED	
2011	Iljazovic E	25	11	Left hip pain	IIA	Debulking, BSO+partialOMT	Ifosfamide, actinomycin, vincristine	10	NED	Bilateral
2011	Aragon L	26	39	Abdominal girth	IV	TAH+BSO	None	3	DOD	
2011	Bosmuller H	27	81	Abdominal pain, abdominal distension	IA	TAH+BSO	AI radiationation	5	NED	Postmenopause
2012	Takahashi H	28	59	Acute abdominopelvic pain, abdominal pain	I	TAH+BSO	Paclitaxel	NR	NR	With dermoid and clear cell carcinoma
2013	Guseh SH	29	40	Fatigue, nausea	IIIC	Optimal debulking	AI	18	AWD	
2013	Albertin C	30	64	Abdominal pain, fever	IIIC	BSO (afterTAH)	Paclitaxel	1.5	DOD	With dermoid
2014	Yaqoob N	31	41	Abdominal pain, bleeding	IA	USO	NR (transfer)	NR	NR	
2014	Yonezawa I	32	29	Abdominal pain, fever	IV	USO	Cysplatine, gemcitabine	84	NED	c-Myc overexpression
2014	Wu PC	33	45	Cyst	IIIA	BSO	MAID	31	DOD	Bilateral with dermoid
2016	Khunamornpong S	34	45	Abdominal distension	IC	TAH+BSO+OMT+LND+apendectomy	AI	NR	NR	With mucinous cystadenocarcinoma
2017	Kudela E	4	44	Abdominal pain, abdominal distension, weight loss	Estimated III	BSO+OMT	None	2	DOD	Bilateral with dermoid
2018	Thankamony P	35	11	Abdominal pain, abdominal distension, fever, vomiting	II	TLH	NR	NR	NR	
2019	Pariury H	36	11	Abdominal pain, abdominal distension	III	USO+biopsies	AI+DG adjuvant: DG hyperthermic intraperitoneal chemotherapy (HIPEC)	43	NED	
2021	Ye H	2	47	Cyst	IA	TAH+BSO	Radiationation olaparib, antiPD-1	9	NED	PD-L1 expression
2021	Peng X	37	25	Abdominal distension	Estimated I	Ovarian biopsies	None	<1	DOD	With dermoid during pregnancy (24 weeks)
2023	Zhou Y	38	51	Abdominal pain, hematuria	IIIC	TAH+BSO+LND	MAID apatinib	<1	DOD	c-Myc overexpression
			41	Fatigue, nausea	IA	TAH+BSO	MAID nivorumab	27	NED	PD-L1 expression
2023	Karpeh MS	3	29	Found incidentalLND on CT	IA	BSO (afterTAH)	Olaparib	20	NED	Somatic pathologic BRCA2 expression
2024	Our case		68	Abdominal pain, anemia	IC3	USO	None	<2	DOD	Postmenopause

Review of the case literature

For the reported cases, the age at onset ranged from 11 to 81 years (mean age, 34 years), and most patients were premenopausal (89.6%). Cutaneous AS, the most frequent type of AS, occurs after radiotherapy for head and neck or breast cancer in older adult patients, whereas patients with primary OAS are relatively young. A previous study suggested that progesterone exacerbated OAS by inhibiting lymphocyte activity, immune response, and T-cell response, and that AS occurred mostly in young women, probably due to the unique effects of sex hormones [37].

However, further case studies on the effects of hormones on primary OAS are needed. Twenty-seven of the cases reported (56.3%) were of advanced disease (stages III and IV), similar to the frequency of typical ovarian cancer. However, the mean overall survival period of stage III and IV cases, which were followed up to death, was 9.14 months, indicating a very poor prognosis. The five-year survival rate for all stages is 10%-35% [39], and the prognosis is poor even for stages I and II. In the present case, the patient was diagnosed with stage IC3 disease. However, peritoneal dissemination and lung metastasis occurred in less than two months, and the disease rapidly progressed to stage IV equivalent disease. Furthermore, hematomas resembling blood blisters occupied the intra-abdominal cavity with progressive peritoneal dissemination, and each one caused intra-abdominal bleeding due to failure to resolve. Three cases of similar hematomas were observed intraoperatively [13,26,32], and two cases of death due to similar rapid intraperitoneal bleeding have been reported [22,37]. This presentation may be due to the presence of abundant vascular cavities within AS, leading to frequent hemorrhages and hematogenous metastases [37]. Therefore, prompt treatment after diagnosis and establishing an appropriate treatment plan are important regardless of the stage of the disease; cases with rapid progression should be treated considering malignancy. In addition, 14 cases have been reported to be associated with other ovarian tumors, and the longest survival period was 30 months. In particular, nine cases of OAS arising from mature teratoma (mean age, 40.1 years; mean overall survival period, 11.4 months) have been reported [4], which may have a poorer prognosis than OAS that occurs alone. Although it has been proposed that OAS may develop due to the malignant transformation of mature teratoma, the mechanism of occurrence and the cause of poor prognosis remain unknown. None of the 47 patients received preoperative chemotherapy, and surgical tumor reduction was the first choice of treatment in all cases.

Treatment options

A summary table of the major cytotoxic chemotherapies and molecular targeted therapies is included (Table 2) [40-48].

**Table 2:** Major cytotoxic chemotherapy and molecularly targeted therapy The table summarizes the treatments considered effective for AS or STS. PD-1: Programmed cell death 1, VEGF: vascular endothelial growth factor, PDGF: platelet-derived growth factor, CTLA: cytotoxic T-lymphocyte antigen, ORR: objective response rate, PFS: progression-free survival, PFI: progression-free interval, OS: overall survival, AEs: treatment-related adverse effects,  irRC: immune-related response criteria, DCR: disease control rate

Chemotherapy classification		Medicine	Explanation of drugs	Year	Author	Reference no.	Target disease	Study design	Endpoints	Result/conclusion
Cytotoxic chemotherapy		Doxorubicin Ifosfamide (AI)	Doxorubicin: anthracycline antibiotics Ifosfamide: alkylating agents	2008	Pervaiz N	40	Localized resectable soft-tissue sarcoma	The paper is a meta-analysis of all randomized controlled trials that evaluated the impact of adjuvant chemotherapy on soft-tissue sarcoma in adults.	The 4 outcomes that were targeted for analysis were local recurrence, distant recurrence, overall recurrence, and OS.	This meta-analysis confirms the marginal efficacy of chemotherapy in localized resectable soft-tissue sarcoma with respect to local recurrence, distant recurrence, overall recurrence, and overall survival. These benefits are further improved with the addition of ifosfamide to doxorubicin-based regimens.
		Mesna Doxorubicin Ifosfamide Dacarbazine (MAID)	Mesna: a prophylactic medication used to reduce the incidence of ifosfamide-induced hemorrhagic cystitis. Dacarbazine: alkylating agents	1993	Antman K	41	Advanced soft tissue and bone sarcoma	This study was an intergroup phase III randomized study of doxorubicin and dacarbazine with or without ifosfamide and mesna in advanced soft tissue and bone sarcoma.	The primary endpoint was ORR. Secondary endpoints included safety, PFS, OS.	Since the toxicity with the three-drug regimen is strong, for older patients or for low-to intermediate-grade lesions, it is preferable to use doxorubicin and dacarbazine first, and if progression occurs, administer ifosfamide.
Molecularly-targeted therapy	immune checkpoint inhibitors (ICIs)	Pembrolizumab	Monoclonal antibody against PD-1	2017	Tawbi HA	42	Advanced soft tissue and bone sarcoma	This study was a multicenter, two-cohort, open label phase 2 trial of pembrolizumab monotherapy performed at 12 US sites that are members of the Sarcoma Alliance for Research through Collaboration (SARC).	The primary endpoint was ORR. Secondary endpoints included safety, ORR by irRC, PFS, OS, and correlation of PD-L1 expression with clinical benefit.	Pembrolizumab has meaningful clinical activity in undifferentiated pleomorphic sarcoma and poorly differentiated/de-differentiated liposarcoma.
		Ipilimumab Nivolumab Trabectedin	Ipilimumab: a human CTLA-4-blocking antibody Nivolumab: PD-1 binding immune checkpoint inhibitor Trabectedin: a marine-derived alkaloid; destroys cancer cells and destroys growth promoting M2 macrophages in the tumor microenvironment	2023	Gordon EM	43	Advanced soft tissue sarcoma	This was a Phase I/II trial of trabectedin plus ipilimumab and nivolumab for Advanced Soft Tissue Sarcoma.	The phase I endpoint focused on determining the maximum tolerated dose in previously treated patients. Phase II endpoints evaluated best response, PFS, OS, and AEs in previously untreated patients.	The SAINT regimen using safe amounts of ipilimumab, nivolumab and trabectedin is safe and effective as a first-line therapy for advanced soft tissue sarcoma.
	Angiogenesis inhibitors (AIs)	Bevacizumab	VEGF-A-targeting monoclonal antibody	2015	Ray-Coquard IL	44	Metastatic or advanced angiosarcoma	This study was a multicenter, randomized, stratified, noncomparative, open-label phase II. It was to evaluate the administration of paclitaxel alone or paclitaxel combined with bevacizumab, for the treatment of inoperable locally advanced or metastatic angiosarcoma.	The primary outcome measure was PFS at 6 months from trial entry. Secondary outcome measures included tumour response, ORR, PFS, OS in patients who started treatment.	The improvement of paclitaxel activity is unlikely to be the consequence of added bevacizumab. This study suggest that the extracellular blockade of VEGF by a monoclonal antibody, such as bevacizumab, would not interfere with the AS proliferation.
		Pazopanib	An oral angiogenesis inhibitor that targets, among other receptors, VEGF receptors, PDGF receptors, and c-kit	2017	Kollár A	45	Advanced vascular sarcoma	A retrospective search of patients with advanced vascular sarcoma treated with pazopanib at multicenter.	The study endpoints were response rate to pazopanib, PFS and OS. The disease control rate was defined as the sum of complete response, partial response and stable disease.	This study documented favorable disease control rate, PFS, and OS with pazopanib, suggesting its activity in AS.
		Apatinib	Selective VEGF receptor-2 tyrosine kinase inhibitor	2017	Li F	46	Advanced sarcoma (stage IV)	This study was single center open label study and evaluated the efficacy and safety of Apatinib for sarcoma patients in stage IV.	PFS, OS, ORR, DCR and AEs were reviewed and evaluated.	This study provides that Apatinib exhibits objective efficacy in stage IV sarcomas with manageable toxicity.
		Olaparib Trabectedin	Olaparib: poly(ADP-ribose) polymerase inhibitor Trabectedin: a marine-derived alkaloid	2018	Grignani G	47	Advanced soft tissue sarcoma	This study was open-label, multicenter, phase 1b trial with advanced soft-tissue sarcoma.	The primary endpoint was determination of the recommended phase 2 dose. (The maximum tolerated dose)	This study showed that the combination had a higher activity in terms of both disease control and PFC. This was observed in those patients with high tumour basal PARP1 expression.
		Axitinib	An oral tyrosine kinase inhibitor targeting VEGFR-1, VEGFR-2, VEGFR-3, PDGF receptor-β and KIT	2023	Woll PJ	48	Advanced soft tissue sarcoma	Axi-STS was a pathologically stratified, non-randomized, open-label, multi-center, phase II clinical trial of oral axitinib in patients with advanced soft tissue sarcoma.	The primary outcome measure was PFS. Secondary outcome measures included tumour response, best percentage change in size of target lesions, PFS, PFI, OS, changes in WHO performance status, and serious AEs.	This study has demonstrated that axitinib has activity in angiosarcoma. However, it was not able to determine the mechanism for this.

Cytotoxicity Chemotherapy

No clear consensus on postoperative adjuvant chemotherapy or remission induction therapy exists. Anthracyclines and ifosfamide constituted the most common chemotherapeutic regimen in 16 of the 24 cases (66.7%) in which the chemotherapeutic agents could be confirmed because these agents have been proven effective for treating metastatic soft tissue sarcomas (STSs), including AS [24,40]. Five of these patients received mesna, doxorubicin, ifosfamide, and dacarbazine (MAID) therapy. Although MAID therapy is associated with severe myelosuppression, a high response rate of approximately 50% has been reported in patients with advanced OAS [3,19,41]. Other commonly used agents include gemcitabine, docetaxel, paclitaxel, and cisplatin.

Molecularly Targeted Therapy

Immune checkpoint inhibitors (ICIs) have also been reported to be effective against STS, with the pembrolizumab study and the ipilimumab plus nivolumab and trabectedin study being effective in advanced STS, but only for specific target sarcoma, and AS was not included as a target disease hemAS was not included as a target disease [42,43]. Although there have been reports of responses to PD-L1 inhibitors, which are ICIs [2,36], there are no large studies on AS. Therefore, it is expected that more cases will be accumulated in the future.

In the field of angiogenesis inhibitors (AIs), a phase II trial recently reported the efficacy of axitinib, a tyrosine kinase inhibitor, in cases of advanced STSs. Axitinib targets VEGFR-1, VEGFR-2, VEGFR-3, PDGFR-β, and KIT and is expected to inhibit angiogenesis extensively; the results of a phase III trial are currently pending (axi-STS trial) [48]. However, bevacizumab, a VEGF inhibitor, has been reported to have no additive effect on treatment for AS with paclitaxel. Genome analysis of AS has revealed the involvement of various vascular growth factors, such as PTPRB and PLCG1, which are different from VEGF, and bevacizumab, a monoclonal antibody, may be unable to control the progression of AS [44]. In a retrospective study of advanced AS, it was reported that pazopanib treatment resulted in a disease control rate of 40%, a median PFS of three months, and a median OS of 9.9 months for AS patients. This suggests that pazopanib has activity in AS [45]. Despite promising findings, there have been no primary ovarian cases reported thus far, and the efficacy of the treatment in this context remains undetermined. There are also reports indicating that the apatinib study and the combination study of olaparib and trabectedin are effective in STS. However, among the eligible patients, the number of patients with AS was 0 and one, respectively, and thus these reports cannot be said to guarantee efficacy in AS.

Treatment for Genetic Mutations

However, the efficacy of treatment based on the genetic background of the tumor has also been suggested, with a report showing the efficacy of gemcitabine in cases of overexpression of the c-Myc gene [32]. The c-Myc gene (somatic lineage) is expressed only in secondary AS in the breast [49]. Among primary OAS cases, differential expression of the c-Myc gene was observed, suggesting its influence on the response to chemotherapy. Similarly, long-term survival with PARP inhibitors for somatic lineage BRCA mutations has been reported [3].

Future prospects

In addition to conventional drug therapy, molecular targeted therapies and immune checkpoint inhibitors may be used in the future for malignant soft tissue tumors. Furthermore, more and more studies are combining AI and ICI in the STS field [50]. Therefore, it is highly likely that similar treatment methods will be established for AS in the future. To achieve this, it is essential to collect cases of the rare disease AS and establish appropriate treatment methods through multifaceted approaches, including comprehensive analysis by next-generation sequencing. The accumulation of cases is highly desirable.

Limitations

In this study, we demonstrated that some cases of primary OAS progress rapidly, and bleeding was difficult to control. However, a limitation of our approach to this case was that we were unable to reach a point where chemotherapy could be administered and, therefore, could not gain new insights into chemotherapy for this rare disease.

## Conclusions

Our study provides a comprehensive analysis of past primary OAS cases, compiling the largest and most recent body of literature to date. Primary OAS is an extremely rare disease with a poor prognosis. In cases with rapid progression, as the present case, considering malignant tumors such as OAS and promptly initiating postoperative therapy is essential. Our case demonstrated that prompt postoperative therapy can temporarily control bleeding, but the lack of established drug protocols underscores the necessity for further research and development of effective treatments.

Recently, molecular and immunological analysis of malignant tumors has progressed, and various treatment methods have been established. Primary OAS has the characteristics of both soft-tissue tumors and ovarian cancer, and analysis from both directions may lead to the discovery of new, personalized, targeted therapies. Further accumulation of cases, including genetic analysis, is needed.
